# A synergistic bactericidal effect of low-frequency and low-intensity ultrasound combined with levofloxacin-loaded PLGA nanoparticles on *M. smegmatis* in macrophages

**DOI:** 10.1186/s12951-020-00658-7

**Published:** 2020-07-29

**Authors:** Shuang Xie, Gangjing Li, Yuru Hou, Min Yang, Fahui Li, Jianhu Li, Dairong Li, Yonghong Du

**Affiliations:** 1 State Key Laboratory of Ultrasound in Medicine and Engineering, College of Biomedical Engineering, Chongqing Medical University, No. 1 Yixueyuan Road, Yuzhong District, Chongqing, 400016 China; 2 Chongqing Key Laboratory of Biomedical Engineering, Chongqing Medical University, Chongqing, 400016 China; 3 Department of Respiratory and Critical Care Medicine, the First Affiliated Hospital of Chongqing Medical University, No. 1, Youyi Road, Yuzhong District, Chongqing, 400016 China

**Keywords:** Levofloxacin-loaded nanoparticles, Low-frequency and low-intensity ultrasound, Bactericidal effect, *M. smegmatis*, Macrophages

## Abstract

**Purpose:**

Tuberculosis (TB) is a highly infectious disease caused by *Mycobacterium tuberculosis* (*Mtb*), which often parasites in macrophages. This study is performed to investigate the bactericidal effect and underlying mechanisms of low-frequency and low-intensity ultrasound (LFLIU) combined with levofloxacin-loaded PLGA nanoparticles (LEV-NPs) on *M. smegmatis* (a surrogate of *Mtb*) in macrophages.

**Methods and results:**

The LEV-NPs were prepared using a double emulsification method. The average diameter, zeta potential, polydispersity index, morphology, and drug release efficiency in vitro of the LEV-NPs were investigated. *M. smegmatis* in macrophages was treated using the LEV-NPs combined with 42 kHz ultrasound irradiation at an intensity of 0.13 W/cm^2^ for 10 min. The results showed that ultrasound significantly promoted the phagocytosis of nanoparticles by macrophages (*P *< 0.05). In addition, further ultrasound combined with the LEV-NPs promoted the production of reactive oxygen species (ROS) in macrophage, and the apoptosis rate of the macrophages was significantly higher than that of the control (*P *< 0.05). The transmission electronic microscope showed that the cell wall of *M. smegmatis* was ruptured, the cell structure was incomplete, and the bacteria received severe damage in the ultrasound combined with the LEV-NPs group. Activity assays showed that ultrasound combined with the LEV-NPs exhibited a tenfold higher antibacterial activity against *M. smegmatis* residing inside macrophages compared with the free drug.

**Conclusion:**

These data demonstrated that ultrasound combined with LEV-NPs has great potential as a therapeutic agent for TB.
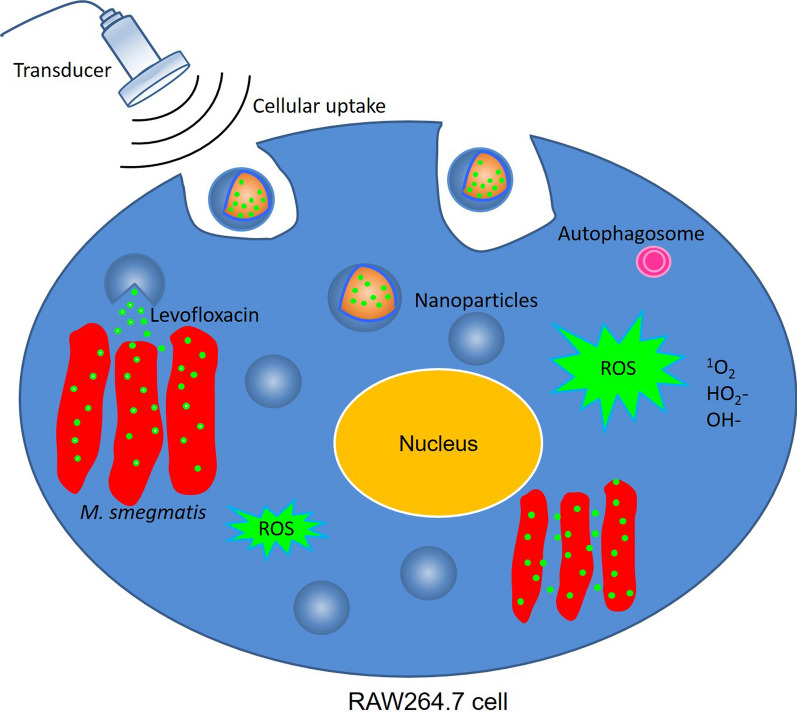

## Introduction

Tuberculosis (TB) is an infectious disease that is a primary cause of ill health. It is one of the top 10 causes of death in the world, in addition to being the number 1 single infectious disease killer (ranking above HIV/AIDS) [1, 2]. Tuberculosis (TB) is caused by the pathogen of *Mycobacterium tuberculosis* (*Mtb*) and transmitted through inhalation of aerosolized droplets containing bacilli from an infected individual when coughing or sneezing. The typical manifestation of TB is pulmonary tuberculosis, but it can also be seen in other sites, such as extrapulmonary tuberculosis [3–5]. According to the World Health Organization, 1.4 million people died of TB, and 10 million new cases were reported in 2019 [1]. *Mtb*, an intracellular bacterial pathogen, is characterized by a thick cell wall and poor permeability, which makes it difficult for drugs to diffuse into *Mtb*. Systemic chemotherapy is currently available as a TB treatment [6]. Compared with other bacterial infections, the treatment period for tuberculosis is prolonged, and it requires more than 6 months of treatment. This long, intensive, and high systemic exposure has many side effects and is one of the primary causes of poor patient compliance or nonadherence with prescribed treatments. This leads to the significant emergence of drug-resistant strains. The treatment of multidrug-resistant tuberculosis patients requires more expensive and toxic drugs that have resulted in poor outcomes [7, 8].

The intracellular survival of *Mtb* itself in macrophages plays a central role in the pathogenesis of TB, which limits the bioavailability of dosed antibiotics in the target area. One of the recent developments in antibacterial strategies in addressing these challenges lies in exploring antimicrobial nanoparticles and antibiotic delivery systems as new tools to tackle the current challenges in efficient antibiotic delivery and to reduce drug toxicity [9–11]. TB patients will benefit from the development of new TB drugs, treatment regimens, and treatment modalities.

The application of nanoparticles as drug and bioactive active molecular carriers has shown attractive potential in disease treatment applications during recent years to achieve the controlled release of drugs [12–14]. Several types of nanoparticles made of various polymers have been designed for use in drug encapsulation to target pathogenic bacteria [15–17]. Among these materials, poly(lactic-co-glycolic acid) (PLGA) is Food and Drug Administration approved for human use, and PLGA particles are the most widely applied type of particles due to their biocompatibility and biodegradability. The extensive use of PLGA nanoparticle-based drug delivery systems is promising in the field of antimicrobial infection due to their higher efficiency and fewer adverse effects [18].

Low-frequency and low-intensity ultrasound (LFLIU) is a novel and noninvasive method for the reversible, selective, and safe application of chemotherapy drug delivery [19, 20]. Ultrasound-induced increases drug penetration into cells is believed to result from oscillations in gas bubbles in the media [21]. These oscillations cause cavitations and disruptions close to the cell surface that shear the membrane, making them more permeable to small molecules and thus allowing increased drug diffusion [22]. Recent studies have shown that LFLIU had been widely adopted by medical researchers to improve the bactericidal effect of antibiotics against planktonic bacteria, bacterial biofilms, chlamydia, and other organisms in vitro and in vivo [23–25]. Numerous studies in the field of ultrasound-mediated intracellular delivery of drugs have demonstrated that the application of ultrasound has an improved efficacy for free drugs and antibiotics encapsulated in nanoparticles. Our previous research demonstrated that LFLIU can effectively enhance the permeability of the cell wall of *M. smegmatis*, thereby enhancing the bactericidal effect of the antibiotic, levofloxacin, on *M. smegmatis* [26]. Our previous work also demonstrated that the synergistic antifungal efficacy of LFLIU combined with amphotericin B-loaded PLGA nanoparticles on *C. albicans* infection was successfully demonstrated using in vitro and in vivo assays [27, 28].

To design more effective strategies against *Mtb*, PLGA nanoparticles encapsulating a conventional anti-TB drug (levofloxacin) are developed in this study. Then the combined effect of LFLIU and drug-loaded nanoparticles against intracellular *M. smegmatis* bacteria is observed based on an in vitro macrophage infection model. It is well known that *M. tuberculosis* (*Mtb*) grows very slowly and is highly contagious and pathogenic. Therefore, laboratories must meet the requirements of the National Biosafety Level 3 (BSL-3) in order to conduct numerous viable *Mtb* operations. *M. smegmatis* is a simple model that is easy to work with in that it has a fast doubling time and only requires a biosafety level 1 laboratory. In addition, it is important that this species shares more than 2000 homologous genes with *M. tuberculosis* and shares the same peculiar cell wall structure of *M. tuberculosis* and other mycobacterial species [29]. Thus, it is often used as a surrogate model for many TB studies [30–32]. Therefore, *M. smegmatis* was selected as the experimental strain in this experiment. In this study, the bactericidal effect and mechanism of LFLIU combined with levofloxacin-loaded PLGA nanoparticles on *M. smegmatis* in macrophages is investigated. The results support the potential of LFLIU combined with drug-loaded nanoparticles as a new, non-invasive, safe, and effective method for the treatment of TB.

## Materials and methods

### Chemicals

Polyvinyl alcohol (PVA), 3-(4,5-dimethyl-2-thiazolyl)-2,5-diphenyl-2-H-tetrazolium bromide (MTT), levofloxacin (LEV), isopropanol, dichloromethane (DCM), dimethyl sulfoxide (DMSO), sodium dodecyl sulfate (SDS), and Tween-80 were purchased from Sigma-Aldrich (St. Louis, MO, USA). Poly (lactide-co-glycolic) acid (PLGA) polymer material with a molecular weight of 21 kDa (ratio of lactide to glycolic acid molar ratio of 50:50) was purchased from Rui Jia Biological (Xi’an, China). Dulbecco’s Modified Eagle’s Medium (DMEM), fetal bovine serum (FBS), trypsin, and phosphate buffered saline (PBS) were obtained from Thermo Fisher Scientific (Waltham, MA, USA). Penicillin–streptomycin solution, a reactive oxygen species (ROS) assay kit, 1,1′-dioctadecyl-3,3,3′,3′-tetramethylindocarbocyanine perchlorate (DiI), and 4,6-Diamidino-2-phenylindole (DAPI) were obtained from Beyotime Biotechnology Co., Ltd. (Shanghai, China). Middlebrook’s 7H9 broth medium, Luria–Bertani (LB) broth, and oleic acid-albumin-dextrose-catalase (OADC) were purchased from BD Biosciences (New York, USA).

### Cell and bacterial culture experiments

Mouse peritoneal macrophages RAW264.7 were purchased from the Shanghai Institute of Cells, Chinese Academy of Sciences and cultured in a humidified incubator under a setting of a partial pressure of 5% CO_2_ at 37 °C in DMEM, which was supplemented with 1% penicillin/streptomycin and 10% FBS. The RAW264.7 cells were generally seeded in a cell culture flask (Corning, USA) for 12 h to adhere. Then, they were harvested using a 0.25% trypsin–EDTA solution for 2 min for the following experiments.

The bacterial strain used in this study was *M. smegmatis* mc2155 (purchased from the National Institute for the Control of Pharmaceutical and Biological Products, Beijing, China), an acid-fast bacterial species, which is considered to be a model organism for researching *Mtb* in the laboratory [29]. The bacteria were grown in Middlebrook’s 7H9 broth medium supplemented with 10% OADC and 0.05% Tween-80 at 37 °C for 24 h with agitation (180 rpm). The bacteria were allowed to reach the exponential phase with an optical density (OD_600_) of 0.6–0.8, and were harvested and re-suspended using PBS to a concentration of 10^6^ CFU/mL for the following experiments. The minimally inhibitory concentration (MIC) of LEV against *M. smegmatis* was determined using the micro-broth dilution method.

### Preparation and characterization of the nanoparticles

The LEV-loaded PLGA nanoparticles (LEV-NPs) were prepared using the double emulsification method by sonication as previously described [28, 33]. Briefly, PLGA was dissolved completely in DCM. A pre-weighed amount of LEV was dissolved in acetic acid that was miscible with water (20:80%, v/v). The dissolved PLGA polymer material and the drug were mixed for the first ultrasonic sonication using an ultrasonic processor (XL2020, USA) in an ice bath at 100 W ultrasonic power for 2 min. Next, the 1% PVA cooled was added to the polymeric mixture for the second ultrasonic sonication in an ice bath at 100 W ultrasonic power for 5 min. After that, 2% isopropanol was added to the suspension, followed by magnetic stirring in an ice bath in the fume hood for at least 4 h until there was no pungent smell to complete the removal of DCM from the prepared nanoparticles. The purity of the prepared nanoparticles was further confirmed using proton nuclear magnetic resonance (1H-NMR) spectroscopy (Varian, Palo Alto, CA, 400 MHz) with no residual peak of the DCM solvent. The LEV-NPs were washed and collected using centrifugation at 8000*g* for 10 min. After that, the LEV-NPs were lyophilized in a freeze dryer (Christ ALPHA 2-4 LSC plus, Osterode, Germany) for the following study. The blank PLGA nanoparticles (blank NPs) and Dil loading PLGA nanoparticles (DiI-NPs) were prepared using a similar method with LEV-NPs, except that the drug solution was exchanged for an equal amount of deionized water or DiI (final concentration of 10 μM).

The average diameter, zeta potential, and polydispersity index (PDI) were determined by dynamic light scattering (DLS) using a Malvern laser particle size analyzer (Zeta SIZER 3000HS, USA). The morphological characterization of the nanoparticles was observed using transmission electron microscopy (TEM, Hitachi High-Technologies, Tokyo, Japan) and scanning electron microscopy (SEM, Hitachi High-Technologies).

### Determination of the LEV-NPs loading content and encapsulation efficiency

A total of 2 mg of freeze-dried nanoparticles was resuspended in 1 mL of dimethyl sulfoxide (DMSO) after the nanoparticles were destroyed. Then the drug concentration was determined using a UV–Vis spectrophotometer (UV-2600 SHIMADZU) at 290 nm. The drug loading content (LC) and encapsulation efficiency (EE) were calculated using the following equations:

$$ {\text{LC }}\left( \% \right) \, = \, \left[ {{\text{weight of the drug in nanoparticles}}/{\text{weight of the nanoparticles}}} \right] \times 100\% ,{\text{ and}} $$

$$ {\text{EE }}\left( \% \right) \, = \, \left[ {{\text{weight of the drug in nanoparticles}}/{\text{weight of the feeding drug}}} \right] \times 100\% . $$

### In vitro ultrasound-triggered LEV release from the LEV-NPs

The kinetic release of LEV from the LEV-NPs in vitro with sonication was investigated. A sample of LEV-NPs lyophilized powder was diluted in PBS by sonication (fixed frequency of 42 kHz) at an intensity of 0.13 W/cm^2^ for 10 min. After sonication, the samples were then individually transferred into dialysis bags (34 mm flat width, MWCO: 7000 Da, Biosharp, Hefei, China), which were then incubated in 50 mL of PBS and shaken at 100 rpm. At each predetermined time point, dialysate samples (1 mL) were individually collected for determination of the LEV concentration using UV–vis spectrophotometry (UV-2600 SHIMADZU, Japan) at 290 nm. Then the samples were returned into the original solution to maintain the total volume of the dialysate constant. Dialysate samples from the LEV-NPs that did not undergo sonication were used as controls. The cumulative drug release (%) was calculated using the following equation:

$$ \begin{aligned} {\text{Cumulative release }}\left( \% \right) \, \hfill \\ {\kern 1pt} \, = \, \left[ {{\text{weight of LEV released from LEV}} - {\text{NPs}}/{\text{initial weight of the drug in LEV}} - {\text{NPs}}} \right] \times 100\% . \hfill \\ \end{aligned} $$

### Ultrasonic irradiation method

**Fig. 1:**
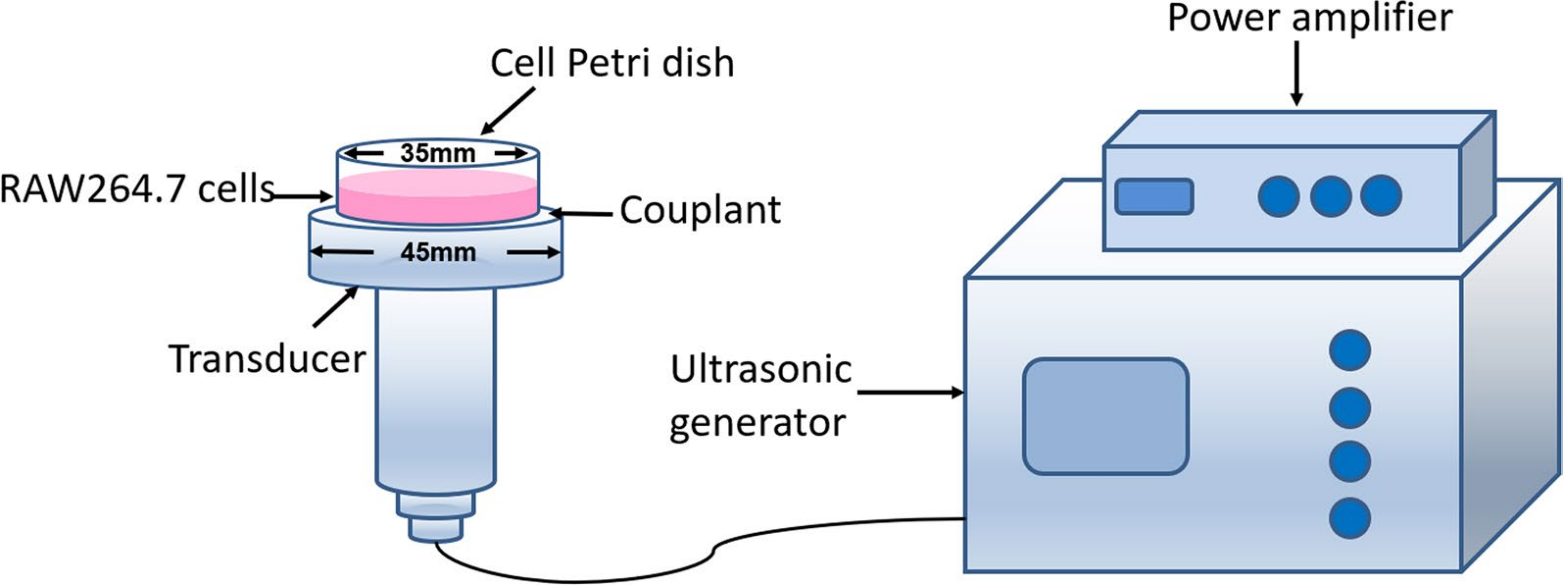
A schematic of the ultrasonic irradiation procedure. The cell Petri dish was placed upright over the center of the transducer (frequency: 42 kHz, diameter: 45 mm)

The LFLIU system device used in this experiment was developed by the Chongqing Medical University Institute of Biomedical Engineering. It had a transducer diameter of 45 mm, a fixed frequency of 42 kHz, and an adjustable ultrasonic intensity output of 0.13 W/cm^2^ to 0.33 W/cm^2^. The acoustic field was measured using a hydrophone (Onda Corp, Sunnyvale, CA, USA). The medical ultrasonic couplant was uniformly coated on the top of the transducer. The 35 mm cell culture dish was placed directly above the transducer and gently squeezed to expel the air. This was followed by ultrasound irradiation (as shown in Fig. 1) with a working mode of a continuous-wave. In this study, the dose of ultrasound at an intensity of 0.13 W/cm^2^ with irradiation for 10 min was selected due to little effect on macrophage activity (based on the preliminary data). Prior to the experiment, all of the samples were equilibrated at room temperature (25 °C) using air conditioners. The temperatures of the cell suspensions were monitored using needle thermo-sensors with digital displays (batch 119, No. 02810232; Yuyao Temperature Instrument Factory Co., Ltd, China).

### Cytotoxicity assay

The cytotoxicity of the LEV and the LEV-NPs against the RAW264.7 cells was investigated using a MTT assay. Briefly, the RAW264.7 cells (1 × 10^5^ cells/mL) were grown in a Petri dish for 24 h to allow cell adhesion. They were then treated with the LEV and LEV-NPs containing the final drug concentrations of 0 μg/mL, 2 μg/mL, 4 μg/mL, 8 μg/mL, 16 μg/mL, 32 μg/mL, 64 μg/mL, 128 μg/mL, and 256 μg/mL for 4 h. After that, the cells were continuously irradiated for 10 min using 0.13 W/cm^2^ ultrasound (the ultrasound dose used was based on the preliminary data), and then the cells were cultured for another 24 h. The MTT experiment was then conducted. The control group consisted of the same procedure, but no ultrasound treatment. The relative cell viability was calculated as follows: cell viability (%) = OD_570_ (treatment)/OD_570_ (control) × 100%. The values are presented as averages of the three independent experiments.

### Phagocytosis of macrophages on the nanoparticles

In this study, DiI-NPs was used as a model to study the phagocytic effect of macrophages on the LEV-NPs under ultrasound. The nucleus of RAW264.7 cells were stained with DAPI (blue fluorescence, 10 μg/mL). DiI-NPs (red fluorescence, 4 μg/mL) were added to the culture dish of RAW264.7 cells, and then irradiated with 0.13 W/cm^2^ ultrasound for 10 min. Those cells without ultrasonic irradiation were used as the control. After incubation for another 3 h, the plates were washed three times to remove the extracellular DiI-NPs. RAW264.7 cells were observed by laser confocal microscopy (CLSM, A1 + R, Nikon, Tokyo, Japan) at excitation/emission wavelengths of 364/454 nm for DAPI and excitation/emission wavelengths of 549/565 nm for Dil. In addition, the relative fluorescence intensity of the intracellular DiI-NPs was quantified by flow cytometry (CytoFLEX, Beckman Coulter, Inc. CA, USA).

### Intracellular killing of *M. smegmatis* and the TEM observations

The killing efficiency of ultrasound combined with nanoparticle treatment on *M. smegmatis* in macrophages was investigated. A total of 10^5^ RAW264.7 cells per well were seeded in a Petri dish and allowed to grow for 24 h. After washing three times with an antibiotic-free medium, freshly cultured *M. smegmatis* (ratio of bacteria/cells: 10:1) were added into the Petri dish to infect the cells for 2 h. They were then washed three times to remove the extracellular bacteria. The RAW264.7 cells infected with *M. smegmatis* alone without ultrasound treatments were used as the controls. Subsequently, the infected macrophages were incubated in DMEM to expose them to the LEV-NPs (where the LEV was at a final concentration of 4 μg/mL) and the free LEV (at a final concentration of 4 μg/mL). After this, the macrophages were irradiated using an ultrasonic intensity of 0.13 W/cm^2^ for 10 min, cultured for another 3 h at 37 °C in an atmosphere of 5% CO_2_, and then the extracellular free LEV and LEV-NPs were washed using PBS. After treatment for 12 h, the cells were washed and collected. One portion of the cell samples after treatment were made into ultrathin sections for observation of the internal structure of the macrophages by transmission electron microscopy (TEM, JEM-1400PLUS, Hitachi High-Technologies). The other portion of the cell samples were lysed using distilled water containing 0.25% SDS for the observation of the intracellular bacteria and an evaluation of bacterial activity. The survival of the intracellular bacteria was estimated by plating serially diluted cultures on 7H10 plates, and the colony-forming units (CFUs) were enumerated after 48 h. All of the samples were plated in triplicate, and the values were averaged from three independent trials. Similarly, the intracellular bacteria were also made into ultrathin sections for TEM observation.

### Quantification of the intracellular reactive oxygen species

The intracellular ROS were analyzed using a flow cytometer (CytoFLEX, Beckman Coulter, Inc. CA, USA) with a ROS reagent kit and fluorescent probe 2′,7′-dichlorodihydrofluorescein diacetate (DCFH-DA), which is the most widely used fluorescent probe for the measurement of intracellular ROS [34]. The DCFH-DA itself has no fluorescence, and after entering the cell, it was hydrolyzed by the esterase in the cell to form dichlorodihydrofluorescein (DCFH). The intracellular ROS oxidizes non-fluorescent DCFH to produce fluorescent dichlorofluorescein (DCF), which is impermeable to the cell membrane. Therefore, the level of ROS in the cells can be known by detecting the fluorescence of the DCF. Briefly, infection RAW264.7 cells were incubated with DCFH-DA (final concentration of 10 μM) for 30 min. The nucleus was blue stained with DAPI (10 μg/mL) for 10 min. After this, the cells were washed and incubated in DMEM for exposure to the LEV-NPs (drug concentration of 4 μg/mL) and the free LEV (4 μg/mL) and treated with ultrasound at an intensity of 0.13 W/cm^2^ for 10 min. Then the treated cells were collected, resuspended in serum-free medium, and measured using the flow cytometer with the excitation setting at 488 nm. The obtained data were analyzed using Cell Lab Quanta SC MPL Analysis software (CytoFLEX, Beckman Coulter, CA, USA). In addition, the level of intracellular ROS production was observed using a laser confocal microscope (CLSM, A1 + R, Nikon) at the excitation/emission wavelengths of 364/454 nm for the DAPI and the excitation/emission wavelengths of 488/525 nm for the DCF. Without ultrasound irradiation, the others were treated with the same method as a control. The experiments were repeated independently three times.

### Apoptosis and necrosis of the RAW264.7 cells

The Annexin V-FITC/PI double staining kit was used to detect the apoptosis and necrosis of RAW264.7 cells under the different treatments: control (no drug, no ultrasound), free LEV(only LEV), ultrasound (US), ultrasound combined with free LEV (US + LEV), LEV-NPs, and ultrasound combined with LEV-NPs (US + LEV-NPs). The drug concentrations in the LEV group and the LEV-NPs group were 4 μg/mL based on the MIC. The ultrasonic dose used was 0.13 W/cm^2^ for 10 min (based on preliminary data). After the treatment was completed, the cells were incubated for another 24 h. The treated cells were collected and resuspended in 1 mL PBS while adding 5 μL of Annexin V-FITC and 10 μL of PI. The dye was mixed and incubated at room temperature for 15 min in a dark environment. The resulting samples were detected using flow cytometry (CytoFLEX, Beckman Coulter, Inc. CA, USA). The obtained data was analyzed using Cell Lab Quanta SC MPL Analysis software (CytoFLEX, Beckman Coulter). The experiments were repeated independently three times.

### Statistical analysis

The results were analyzed using a one-way ANOVA in SPSS 17 statistical software (IBM, Chicago, USA). The data were expressed as mean ± standard deviation. A *P* < 0.05 was considered to be statistically significant.

## Results

### Characterization of nanoparticles

**Fig. 2:**
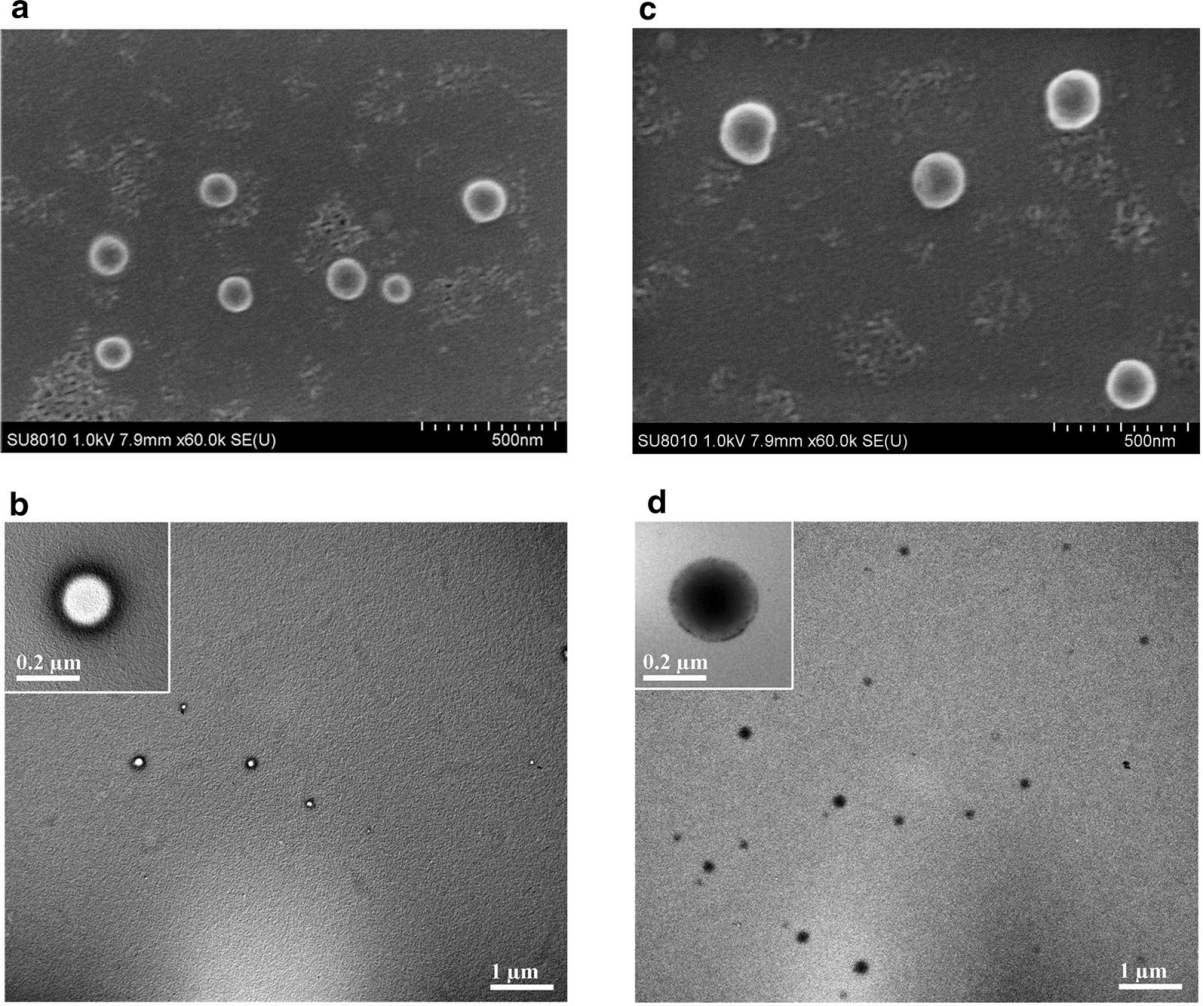
SEM and TEM images of the blank NPs and LEV-NPs. **a** SEM image of the blank NPs; **b** TEM image of the NPs; **c** SEM image of the LEV-NPs; and **d** TEM image of the LEV-NPs

The physical properties of the blank NPs and LEV-NPs are presented in Fig. 2. SEM imaging indicated that the blank NPs and LEV-NPs exhibited a smooth and uniform spherical morphology (Fig. 2a and c). The center of the blank NPs was bright under TEM, while the LEV-NPs showed an enhanced dark area (Fig. 2b and d). In particular, at lower magnifications, the TEM image for the LEV-PLGA showed only black dots. This phenomenon can be simply explained. In TEM scanning, samples with a higher atomic number will block more electrons and result in a darker image. This proved that the LEV was loaded into the PLGA shell.

Table 1 shows the results of the LC and the EE for the LEV-NPs (8.36 ± 0.74% and 84.74 ± 1.28%, respectively). In addition, the size distribution, zeta potential, and polydispersity index (PDI) of the NPs and LEV-NPs were compared in this study. The average diameter of the LEV-NPs was 229.8 ± 12.1 nm, with a (PDI) of 0.038 ± 0.007, and the nanoparticles surface was negatively charged. The zeta potential is an important factor for nanoparticle stability. A reasonable zeta potential can prevent NPs from aggregation [35]. The particle size of the LEV-NPs was greater than that of the NPs, and the difference was statistically significant (*P* < 0.05). In short, the above results indicate that the LEV-NPs were successfully prepared with a uniform size, and this produced stable properties for the delivery of the LEV.

**Table 1:** Physical characteristics of the developed nanoparticles

Formulations	Particle size (nm)	Zeta potential (mV)	PDI	LC (%)	EE (%)
Blank NPs	173.1 ± 13.2	−20.8 ± 4.25	0.134	–	–
LEV-NPs	229.8 ± 12.1*	−28.8 ± 3.78	0.038	8.36 ± 0.74	84.74 ± 1.28

NPs: nanoparticles; LEV-NPs: levofloxacin-loaded nanoparticles; PDI: polydispersity index; LC: loading content; EE: encapsulation efficiency

Standard deviation for n = 3. *P <0.05 compared with NPs

### In vitro investigations of ultrasound-triggered drug release

**Fig. 3:**
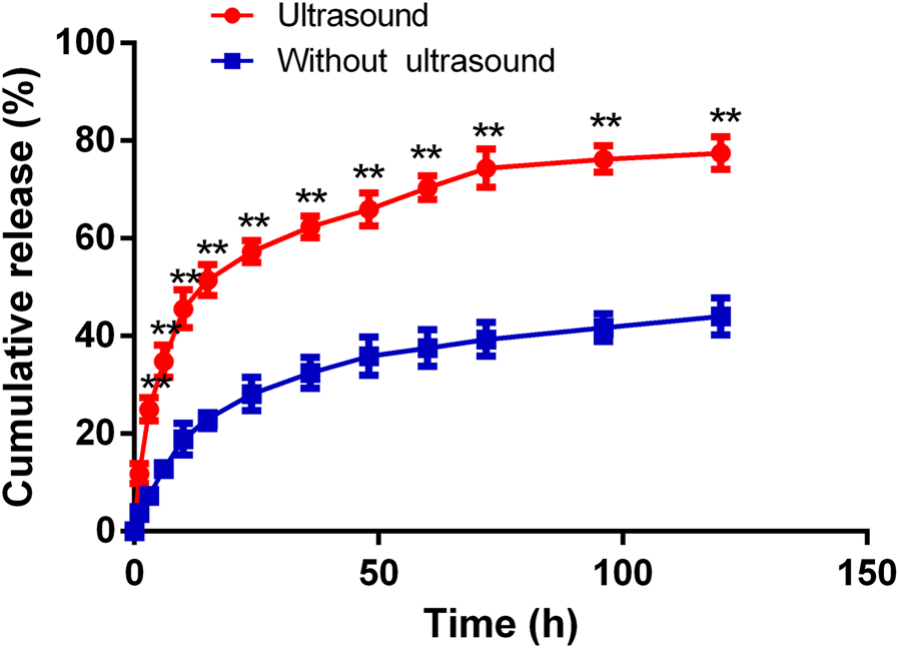
In vitro release profile of the LEV from LEV-NPs with ultrasound and without ultrasound treatment. The experiments were performed in triplicate; mean ± SD are shown. **P < 0.01 compared with those without ultrasonic treatment

The LEV released from the LEV-NPs was assessed by performing in vitro ultrasound-triggered experiments. Figure 3 displays the drug release curves of the LEV-NPs with and without ultrasound. The results showed that the release rate of LEV with sonication was faster than that without sonication. For the LEV-NPs with ultrasound, approximately 74.4% and 77.5% of the LEV were released after 72 h and 120 h, respectively. However, for the LEV-NPs without ultrasound, approximately 39.3% and 44.0% of the encapsulated LEV were released after 72 h and 120 h, respectively. This result implies that the LEV-NPs can be controllably triggered with ultrasound. The PLGA shells may be destroyed by ultrasound to promote the diffusion of LEV, resulting in increased drug release rates.

### Cytotoxicity of the LEV-NPs on macrophage cells

**Fig. 4:**
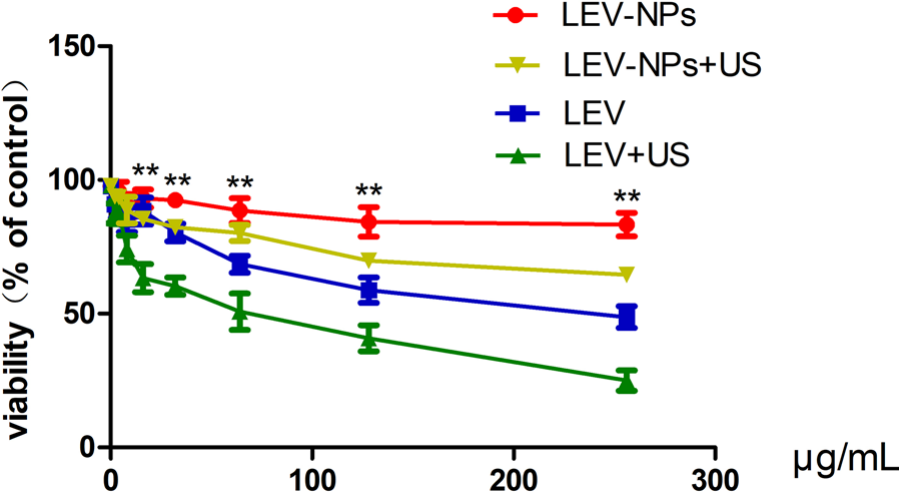
Cytotoxic activity of the LEV-NPs at different drug concentrations on RAW264.7 cells. The cytotoxicity of LEV-NPs to RWA264.7 was measured after RAW264.7 cells were treated with LEV-NPs for 4 h and then cultured for another 24 h. Compared with the LEV + US, ^**^P < 0.05. The experiments were performed in triplicate; mean ± SD are shown

An in vitro cytotoxicity experiment was conducted in the RAW264.7 cells using the MTT assay. Figure 4 shows the cytotoxicity of the free LEV and LEV-NPs with and without ultrasonic treatment on the RAW264.7 cells. The cell viability decreased to 68.52 ± 5.46% when the cells were treated using free LEV alone at a concentration of 64 μg/mL. With the use of the free LEV combined with ultrasound treatment, cell viability began to decline when the concentration of free levofloxacin was 8 μg/mL, which was 74.18 ± 8.58%. For both cases, with an increase in the concentration of the free drug, the decline in cell viability was more pronounced. The LEV-NPs alone at drug concentrations from 0 μg/mL to 256 μg/mL were not cytotoxic to the RAW264.7 cells. When the LEV-NPs were combined with ultrasound treatment, the cell viability began to decrease when the drug concentration in the nano-preparation was 128 μg/mL, which was 69.84 ± 4.26%. It is also obvious that at the same drug concentration and with or without ultrasound, the toxicity of the LEV-NPs on the RAW264.7 cells was lower than that of free drugs.

### LFLIU promoted phagocytosis of drug-loaded nanoparticles

**Fig. 5:**
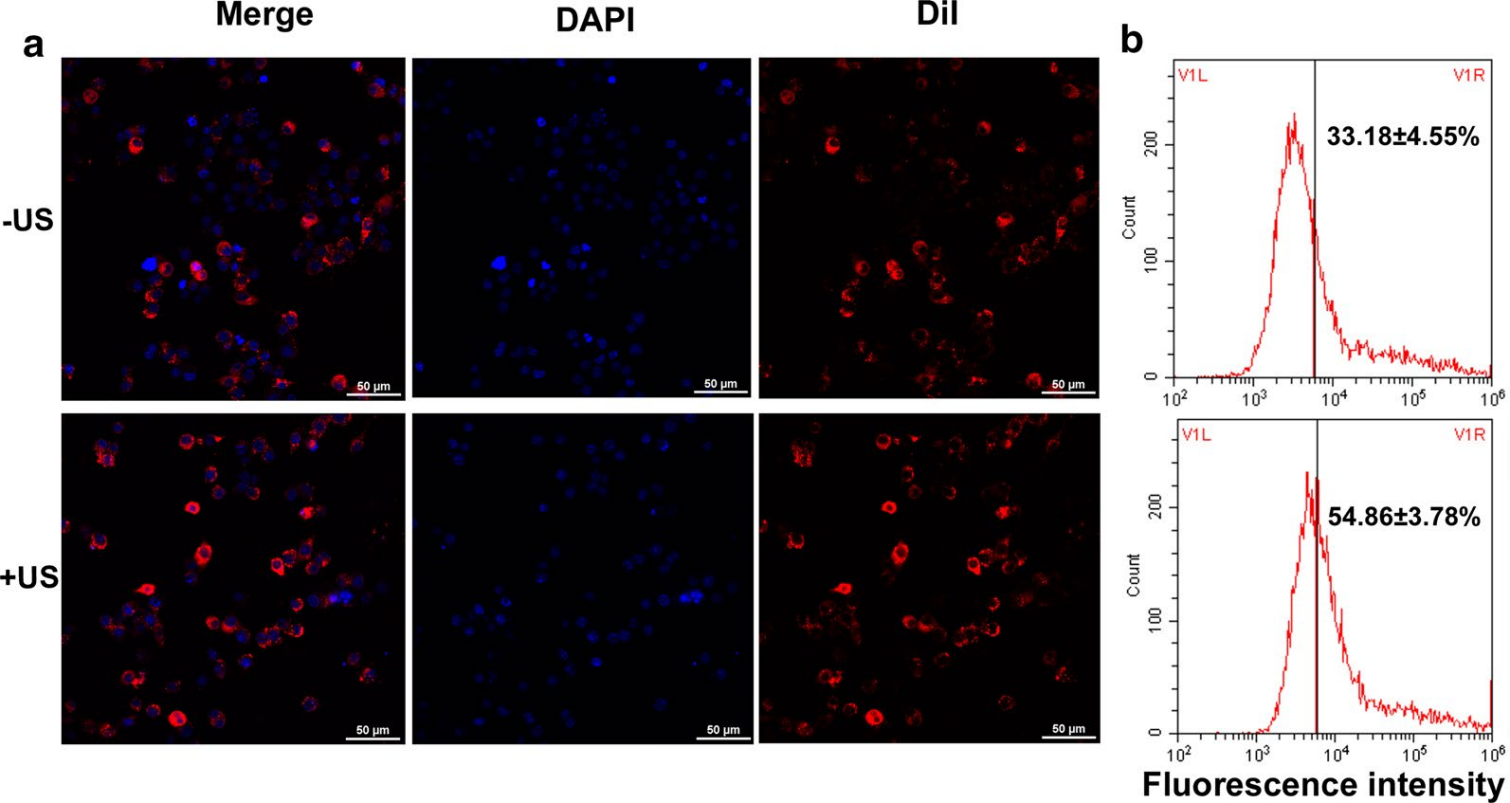
Cellular uptake of Dil-NPs by the RAW264.7 cells. **a** Intracellular Dil-NPs (red) were observed by laser scanning confocal microscopy, amplification ×400. **b** The fluorescence intensity of the intracellular nanoparticles was detected using flow cytometry. Experiments were performed in triplicate; mean ± SD are shown. A *P* < 0.05 was used to compare the fluorescence intensity of intracellular nanoparticles between the US + Dil-NPs group and the Dil-NPs group

Dil-NPs (red fluorescence) were used as a model to evaluate the macrophage phagocytosis to the nanoparticles. The red fluorescence intensity of the intracellular DiI-NPs was observed following the different treatments. Figure 5 shows the phagocytosis of the DiI-NPs after the RAW264.7 cells were treated with DiI-NPs or combined with ultrasound. A laser confocal microscope showed that the DiI-NPs (red) were tightly surrounded around the nucleus (blue). The flow cytometry analysis showed that the relative fluorescence intensity of the DiI-NPs group was 33.18 ± 4.16%, while the phagocytosis rate of the ultrasound combined with the DiI-NPs group was 54.86 ± 7.45%. Obviously, the phagocytosis rate of the ultrasound combined with the Dil-NPs group was higher than that of the DiI-NPs group (*P* < 0.05).

### Post-treatment intracellular bacterial vitality

**Fig. 6:**
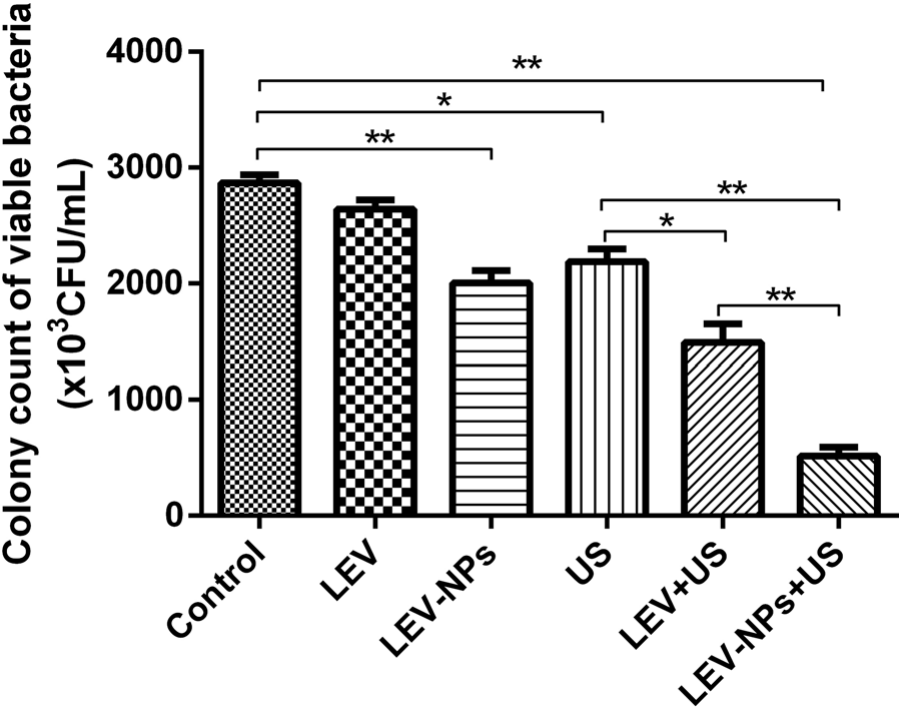
Ultrasound combined with the LEV-NPs exhibited intracellular killing activity against *M. smegmatis*. The bacterial survival rate was determined using a CFU assay. The control group did not undergo ultrasound irradiation and the drug. The experiments were performed in triplicate; mean ± SD are shown. *P < 0.05, **P < 0.01

Fighting intracellular pathogens is a major challenge due to the fact that the cell membrane/wall is a critical barrier to a drug to be used to enter cells to kill bacteria. This is because mycobacterium is an intracellular pathogen, and they are resistant to numerous drugs due to their lipid-rich cell wall [36]. The intracellular burden of *M. smegmatis* in macrophages after being treated with free LEV, LEV-NPs, ultrasound (US), ultrasound combined with LEV (US + LEV), and ultrasound combined with LEV-NPs (US + LEV-NPs) was determined in this study. As shown in Fig. 6, at 12 h after the treatments, US + LEV-NPs, US + LEV, LEV-NPs, and US killed 82.2%, 48.1%, 23.7%, and 30.0% of *M. smegmatis*, respectively, whereas free LEV alone killed 7.8% of the bacteria under similar conditions compared with the control. Obviously, ultrasound combined with the LEV-NPs resulted in an approximate two-fold decrease in the intracellular bacterial burden compared with ultrasound combined with free LEV.

### The cell structure following treatment

**Fig. 7:**
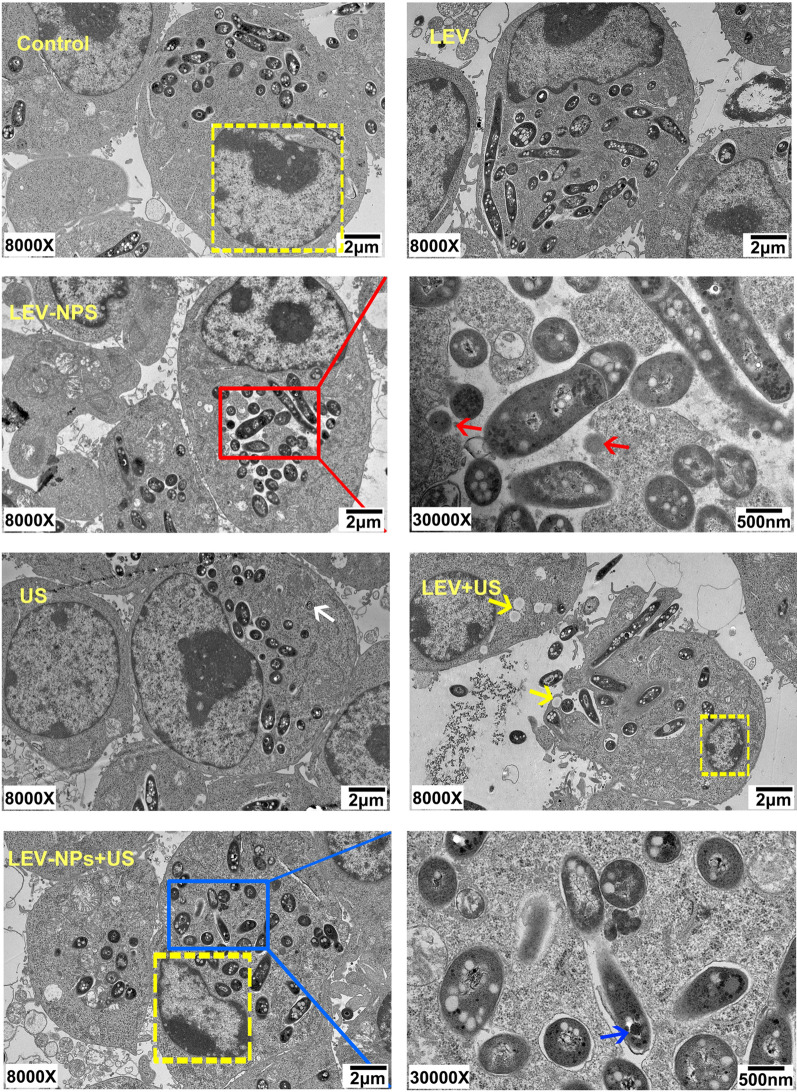
TEM analysis of the morphological change in the RAW264.7 cells and the intracellular *M. smegmatis* in the different treatment groups. Nucleus in the yellow dotted line; autophagosomes (white arrows) were found in the US group; Intracellular nanoparticles (red arrows) were found in the LEV-NPs group (magnification, ×30,000); lipid droplets (yellow arrow) were found in the LEV + US group; Nanoparticles (blue arrows) in bacteria were found in the LEV-NPs + US (magnification, ×30,000)

The TEM observations of the RAW 264.7 cells and intracellular *M. smegmatis* following the different treatments are shown in Fig. 7. The cells in the control were intact with abundant cytoplasm and mitochondria. The cell membranes and the nuclear envelope were intact, and the nuclear materials were uniform. The morphology and structure of the cells in the free LEV group were similar to that in the control. In the LEV-NPs group, the RAW 264.7 cells were injured to some extent, and a small number of cells displayed limited microvilli, the cytoplasm became less dense, and the LEV-NPs (red arrow) could be seen around the nucleus. In the US group, the macrophage structure was relatively intact, but autophagosomes (white arrows) could be observed. In the LEV + US group, the cell morphology was significantly changed, and the cells were severely damaged, a portion of cell microvilli had vanished, the volume of the cell nucleus had decreased, and the chromatin was densely gathered. In addition, lipid droplets (yellow arrow) were clearly visible. In the LEV-NPs + US group, the volume of the cell nucleus had decreased more compared with the other treatments, and the chromatin was condensed in the nuclear envelope, thus presenting the characteristics of apoptosis. It is worth noting that drug-loaded nanoparticles (blue arrows) in *M. smegmatis* were observed.

**Fig. 8:**
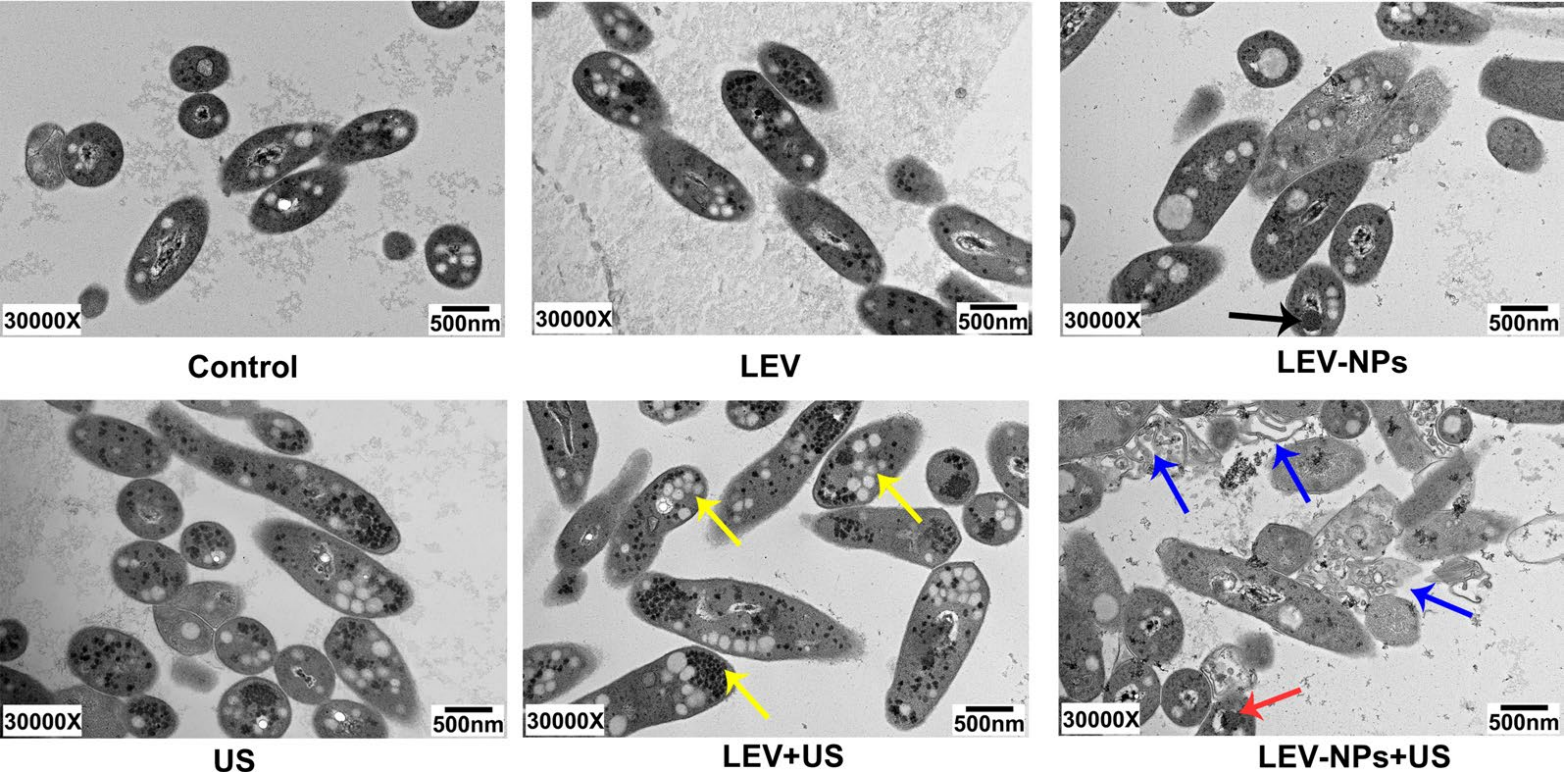
TEM analysis of the damage to *M. smegmatis* in the different treatment groups. The black arrow indicates the LEV-NPs; the yellow arrow indicates lipid droplets and glycogen; the blue arrow indicates the broken cell wall; and the red arrow indicates the LEV-NPs. (magnification, ×30,000)

Figure 8 shows the bacterial damage when *M. smegmatis* was extracted from the lysis of macrophages due to the different treatments. The bacterial cell wall integrity in the control group and the LEV group is shown. In the LEV-NPs group, a portion of the cytoplasm of *M. smegmatis* was not dense, and LEV-NPs (black arrows) within the bacteria were observed. There was no significant change in the bacterial morphology in the US group. However, in the LEV + US group, bacterial swelling, bacterial lipid droplets, and glycogen (yellow arrow) can be seen from the macrophages. In the LEV-NPs + US group, the bacterial cell wall is broken (blue arrow), and cell structure is incomplete, indicating that the cells were severely damaged. In addition, LEV-NPs in *M. smegmatis* were observed (red arrow).

### The intracellular ROS level following treatment

**Fig. 9:**
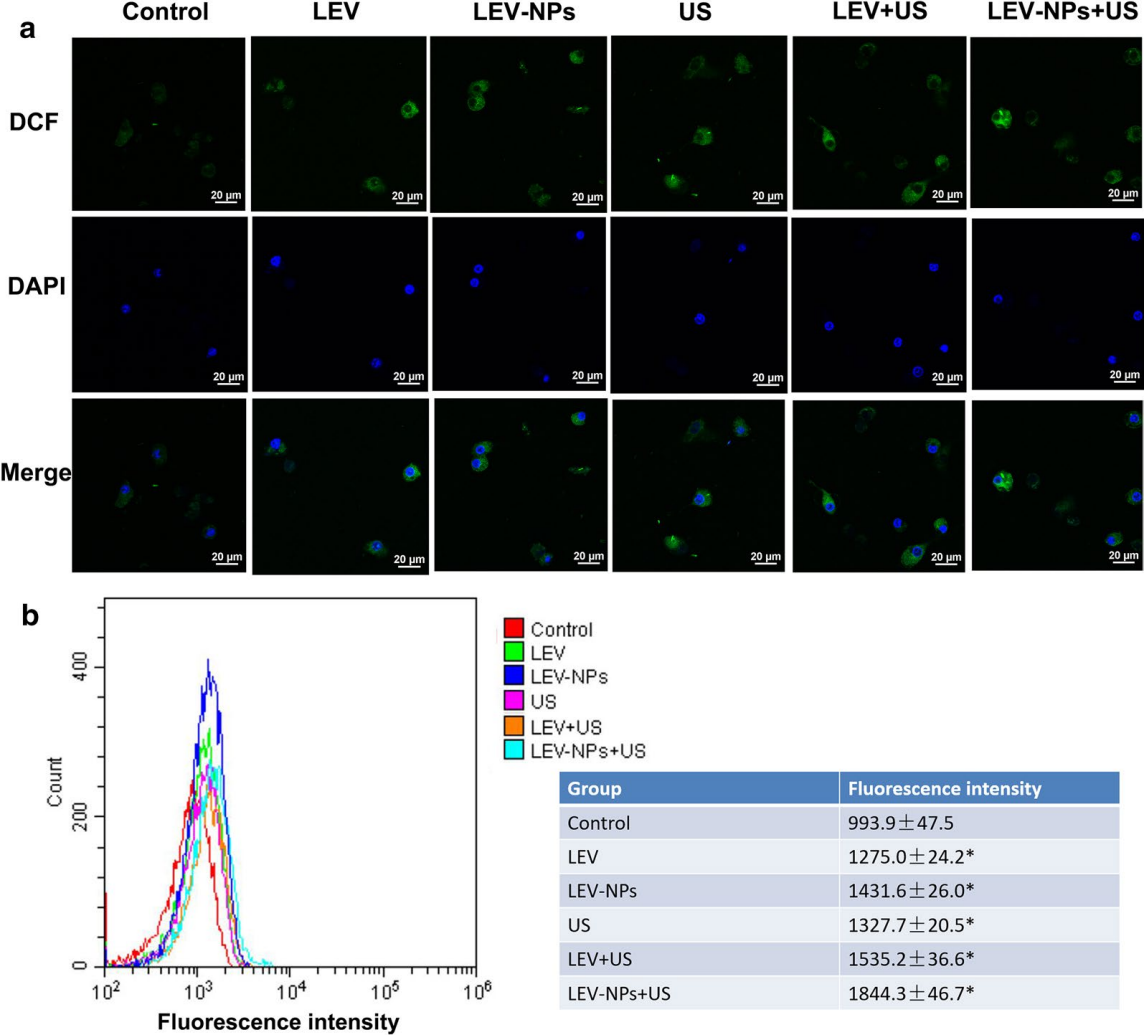
Intracellular ROS production determination. **a** The intracellular ROS (green) of the different treatments was detected by laser confocal microscopy, amplification ×400. **b** The intracellular ROS level in the different treatment groups was analyzed by flow cytometry. The experiments were performed in triplicate; mean ± SD are shown. *P < 0.05 compared with control

The intracellular generation of ROS was observed by laser scanning confocal microscopy and flow cytometry with DCFH-DA (Fig. 9). Figure 9a qualitatively shows that the ROS level (green fluorescence) of the LEV group, the LEV-NPs group, the US group, the LEV + US group, and the LEV-NPs + US group were higher than that of the control group. The results of the quantitative analysis of the ROS level found by flow cytometry, as shown in Fig. 9b, were consistent with those in Fig. 9a. In addition, the fluorescence intensity of the treatment group was higher than that of the control group (*P* < 0.05). The results showed that the green fluorescence intensity of the LEV-NPs + US group was the highest, reaching 1844.3 ± 46.7, which was approximately twice that of the control group (993.9 ± 47.5).

### Apoptosis and necrosis of macrophages following treatment

**Fig. 10:**
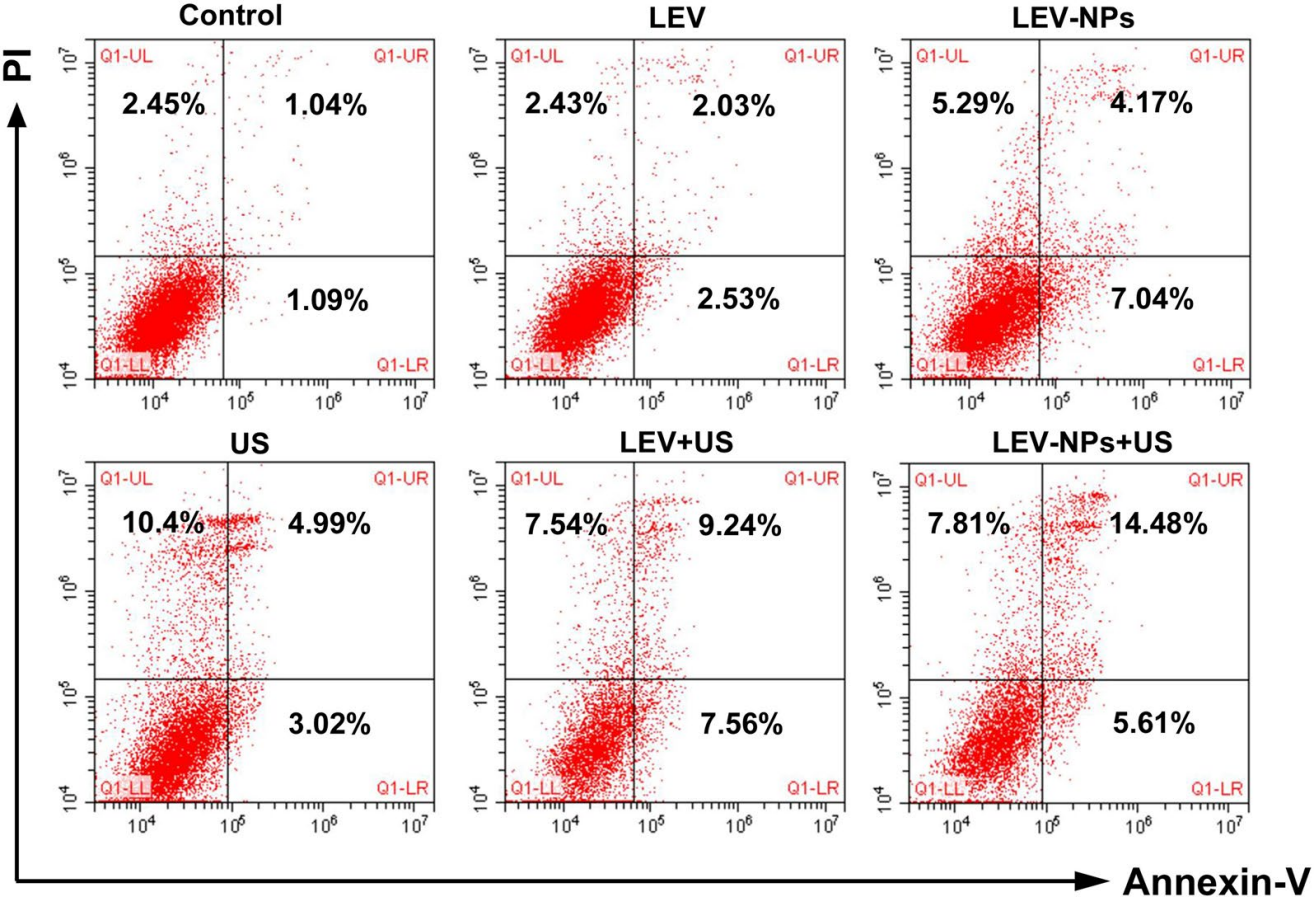
Apoptosis and necrosis ratio of the RAW264.7 cells determined using flow cytometry after double staining with Annexin-V/PI. The upper right quadrant indicates early apoptotic cells; the lower right quadrant indicates late apoptotic cells; the upper left quadrant indicates necrotic cells; and the lower-left quadrant indicates normal cells

The apoptotic rate and necrosis rate of the RAW264.7 cells are shown in Fig. 10 and Table 2. Both apoptosis and necrosis were observed in macrophages, and the apoptotic ratio was higher than the necrotic ratio under the appropriate conditions. Compared with the control group, the apoptosis rates of the LEV group, the LEV-NPs group, the US group, the LEV + US group, and the LEV-NPs + US group increased. The maximum apoptosis ratio (21.25 ± 1.15)  % was observed in the LEV-NPs + US group. Compared with the control group, the necrosis rate of the LEV-NPs group, the US group, the LEV + US group, and the LEV-NPs + US group increased. It is worth noting that there was a nearly equal cell necrotic ratio in the US group, LEV + US group, and LEV-NPs + US group.

**Table 2:** Necrosis rate and apoptotic rate of the RAW264.7 cells under the different treatments

Groups	N	Apoptosis rate	Necrosis rate
Control	6	3.30 ± 1.01	2.21 ± 0.21
LEV	6	6.60 ± 1.78^*^	2.28 ± 0.35
LEV-NPs	6	10.42 ± 0.84^*#^	4.20 ± 1.04^*#^
US	6	8.76 ± 0.78^*^	9.54 ± 0.88*^#^
LEV + US	6	15.54 ± 1.30^*#^	7.82 ± 044^*#^
LEV-NPs + US	6	21.25 ± 1.15^*#^	8.21 ± 0.66^*#^

LEV: levofloxacin; LEV-NPs: levofloxacin-loaded PLGA nanoparticles; US: ultrasonic; SD: standard deviation

Compared with the control, ^*^P < 0.05; compared with the LEV, ^#^*P* < 0.05. Data represent the mean ± SD

## Discussion

A fundamental limitation in the current available regimens for *mycobacteria tuberculosis* is the long duration of therapy that utilize multiple drugs. Nanomedicine has dramatically changed the concept of traditional medicines for treating diseases, and many nanomedicine delivery systems have shown great promise [14, 37]. In a previous study, PLGA nanoparticles that encapsulated RIF and INHP anti-tuberculous agents were synthesized and their antimicrobial activities were determined against intracellular *M. smegmatis* [33]. The results of their in vitro assays suggested that the co-administration of nano-formulated RIF and INHP improved the therapeutic index and drug efficacy compared with native drugs. However, these nano-formulated anti-tuberculous agents delivered the drug to its target using free passive movement, then passively release the drugs to kill the bacteria, which is slow and uncontrollable. This release profile is undesirable because it cannot achieve the minimal drug dosage for maximum patient compliance. Recently, many efforts have been made to develop new drug delivery and release systems. Ultrasound for triggered drug delivery has many advantages over traditional drug delivery methods [38], and the application of ultrasound combined with drug-loaded nanoparticles is a hot topic for many scholars. In recent years, ultrasound combined with drug-loaded nanoparticles has been widely reported for the treatment of tumors, the killing bacteria, and inhibiting biofilm growth [39–41]. In this study, the bactericidal effect and underlying mechanisms of low-frequency and low-intensity ultrasound (Device as shown in Fig. 1) combined with LEV-NPs on *M. smegmatis* in macrophages was investigated.

Biodegradable polymeric nanoparticle-encapsulated formulations are an efficient and promising tool for delivering therapeutic molecules to infected tissue, and this technique is particularly useful to improve the anti-infection effect [42–44]. In this study, levofloxacin-loaded nanoparticles were successfully prepared using a PLGA copolymer utilizing a double-emulsification method. The average particle size was 229.8 ± 12.1 nm, and the size was uniform (see Fig. 2). The cytotoxicity of the LEV-NPs and free LEV with or without ultrasound on RAW264.7 macrophages was compared. The results showed that when the concentration of free LEV reached 8 μg/mL, the cell viability decreased to 68.52 ± 5.46%. With an increase in the drug concentration, damage to cell activity was further aggravated. Nevertheless, the drug concentration in the LEV-NPs reached 64 μg/mL even with ultrasound and showed no cytotoxicity, indicating that the nanoparticles reduced the toxic effect of the drug on the cells (see Fig. 4).

Ultrasound triggered release of LEV from polymeric nanoparticles was investigated in this study. In this experiment, the natural release rate of LEV from drug-loaded nanoparticles in 72 h was only approximately 39.3%, which indicated that it is a long-term slow-release process. However, the drug release rate from nanoparticles increased nearly twice after irradiation with a certain dose of low-frequency ultrasound. The results indicated that low-frequency ultrasound irradiation could promote the release of the contents from drug-loaded nanoparticles (Fig. 3). This result is consistent with earlier studies that demonstrated that low-frequency acoustic activity could stimulate the release of therapeutic substances from nanoparticle formulations, increasing the local concentration of drugs and thereby shortening the treatment period [45].

Previous studies have reported that low-frequency ultrasound (20–100 kHz) produced a series of “acoustic biological effects” dominated by acoustic cavitation, instead of thermal effects. Cavitation events occurring during ultrasound irradiation is the key mechanism of sonoporation [46–48]. Low frequency acoustic cavitation-induced sonoporation offers a noninvasive method of drug delivery. Ultrasonic sonoporation is the temporary opening in the cell membrane/wall, which increases the permeability of the cell plasma membranes and allows for the exchange of substances inside and outside the cell [49, 50]. In addition, nanoparticles may act as cavitation nuclei that enhances the sonoporation effect, which is helpful to increase cell membrane permeability and further drug intake [51, 52].

The results in this study confirmed that the joint use of ultrasound and drug-loaded nanoparticles had a synergetic bactericidal efficacy on *M. smegmatis* in macrophages compared with the other treatments. The action of ultrasound is beneficial to realize the effective delivery of LEV-NPs to *M. smegmatis* in macrophages and increase the drug concentration in the cell to kill intracellular *M. smegmatis*. This experiment regarding the ultrasound promotion of macrophages to uptake nanoparticles (see Fig. 5) also confirmed this argument indirectly. In addition, an increase in lipid droplets and glycogen in *M. smegmatis* was observed, in addition to an incomplete cell wall rupture structure following LEV-NPs + US (Fig. 8). All of these results indicate that ultrasound combined with drug-loaded nanoparticles caused the most serious damage to *M. smegmatis.* This was likely because the rupture of nanoparticles enhanced the acoustic cavitation effect through some complex dynamics during ultrasound irradiation, which further increased the cell wall permeability and more effectively promoted the drug delivery into the cells, thus improving the bactericidal effect.

The TEM images indicated that in the LEV-NPs + US group, the volume of the nucleus decreased and the chromatin condensed, showing the characteristics of apoptosis. this was consistent with the results of a direct analysis using flow cytometry where the highest apoptotic rate was observed (21.25 ± 1.15%) (see Fig. 10 and Table 2) in the LEV-NPs + US group. Ultrasound promoted the apoptosis of macrophages, and the apoptosis of macrophages itself is conducive to the death of intracellular bacteria.

Apoptosis is an innate macrophage defense mechanism. Apoptosis of infected macrophages is associated with diminished pathogen viability. This concept is supported by the finding that apoptosis reduces the viability of *intracellular bacillus Calmette*-*Guerin* and *Mtb* [53, 54]. Necrosis can also be observed in the infected macrophages of some of the treatment groups (Fig. 10 and Table 2). Necrosis is a mechanism used by bacteria to exit the macrophage, evade host defenses, and spread [55, 56]. This seems to be a conflict with the fate of intracellular *M. smegmatis* if apoptosis and necrosis were induced in infected macrophages that underwent the different treatments. In this study, the necrosis ratio in some groups (e.g., LEV-NPs + US, LEV + US) increased slightly, and the apoptosis ratio increased more significantly. These results indicated that the rate of apoptosis induced by ultrasound combined with drug-loaded nanoparticles was more significant than necrotic ratio and helped to decrease intracellular bacterial viability.

In addition to the above mechanism, another possible mechanism of enhanced antibacterial action may be related to an increase in the intracellular activity of reactive oxygen species (ROS). The stimulated generation of ROS after ultrasound irradiation is toxic to microorganisms and has a significant antimicrobial activity against planktonic and biofilm forms [28, 57]. The production of ROS was qualitatively and quantitatively analyzed by confocal laser microscopy and flow cytometry (see Fig. 9) in this study. The intracellular ROS level in the group with the combination of ultrasound and LEV-NPs was the highest among all of the groups.

In summary, the combination of LFLIU and levofloxacin-loaded PLGA nanoparticles produced a significant synergistic bactericidal effect on *M. smegmatis* in macrophages when compared with the standard drugs or drug-loaded nanoparticles alone. The delivery system efficiently translocated the drug into the cell where the pathogens reside and replicate and achieved the maximum therapeutic benefit of the antimicrobial agent. The combined strategy presented here has the following advantages: (1) LFLIU can trigger the release of levofloxacin from nanoparticles, thus increasing the local concentration of drugs; (2) it can promote the uptake of nanoparticles to macrophages; (3) it can promote the apoptosis of macrophages, an innate macrophage defense mechanism; (4) and it can induce levofloxacin to produce reactive oxygen species in macrophages, which are toxic to microorganisms. All of these factors contributed to bacterial damage. The combination of ultrasound with drug-loaded nanoparticles can be considered as a novel and prospective strategy for TB therapy to efficiently achieve drug delivery to effectively kill MTB and is expected to significantly shorten the course of chemotherapy for TB.

## Data Availability

The data, analytical methods, and study materials for the purposes of reproducing the results or replicating procedures can be made available on request to the corresponding author who manages the information.
